# Environmental Enrichment Suppresses Food Seeking and Increases Inhibitory Interneuron Excitability While Decreasing Corticothalamic Neuronal Recruitment in the Prelimbic Cortex

**DOI:** 10.1111/ejn.70315

**Published:** 2025-11-18

**Authors:** Kate Z. Peters, Romarua Agbude, Oliver G. Steele, Nobuyoshi Suto, Eisuke Koya

**Affiliations:** ^1^ Sussex Neuroscience, School of Psychology University of Sussex Falmer UK; ^2^ Department of Clinical Neuroscience, Brighton and Sussex Medical School University of Sussex Falmer UK; ^3^ Department of Molecular Pharmacology and Experimental Therapeutics Mayo Clinic Rochester New York USA

**Keywords:** environmental enrichment, interneurons, motivation, prefrontal cortex, pyramidal cells

## Abstract

Cues such as fast‐food advertisements associated with food can provoke food cravings which may lead to unhealthy overeating. To effectively control such cravings, we need to better understand the factors that reduce food cue reactivity and reveal corresponding ‘anti‐craving’ brain mechanisms. We previously reported that access to environmental enrichment (EE), that provides cognitive and physical stimulation in mice, reduced cue‐evoked sucrose seeking and prelimbic cortex (PL) neuronal reactivity.

To date, the phenotype of PL neurons that undergo EE‐induced adaptations has not been fully elucidated. Therefore, we used brain slice electrophysiology to investigate how EE modulated intrinsic excitability in the general population of PL interneurons and pyramidal cells. Additionally, we used retrograde tracing and the neuronal activity marker ‘Fos’ to investigate how EE modulated cue‐evoked recruitment of pyramidal cells projecting to the paraventricular nucleus of the thalamus (PVT) and nucleus accumbens core (NAc).

Before the cue‐evoked sucrose seeking test, EE increased the general, baseline excitability of inhibitory interneurons, but not pyramidal cells. During cue‐evoked sucrose seeking, EE selectively suppressed recruitment of corticothalamic (PL → PVT), but not corticoaccumbens (PL → NAc), projections. Collectively, these findings advance our understanding of EE's ‘anti–food‐seeking’ actions by demonstrating both cell type‐specific increases in local inhibitory tone and circuit‐specific modulation of PL output pathways.

## Graphical abstract

Environmental enrichment (EE) reduces cue‐evoked sucrose seeking and is accompanied by enhanced inhibitory interneuron excitability and suppressed corticothalamic recruitment in the prelimbic cortex. Using brain slice electrophysiology, retrograde tracing and Fos assays, we show that EE increases baseline excitability of interneurons but not pyramidal cells, and selectively suppresses PL → PVT, but not PL → NAc, projection neurons during cue exposure. These findings reveal cell‐ and circuit‐specific mechanisms underlying EE's anti‐craving effects and modulation of food cue reactivity.

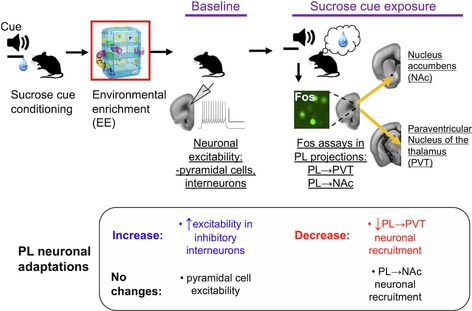

**Abbreviations**
AAV: adeno‐associated virus
AAVDJ: AAV serotype DJ, a hybrid capsid used for efficient transduction
AAVrg: retrograde adeno‐associated virus
aCSF: artificial cerebrospinal fluid
AHP: afterhyperpolarization
AP: action potential or anterior–posterior (for coordinate axis in stereotaxic surgery)
ATP: adenosine triphosphate
BK: big potassium (large conductance calcium‐activated potassium) channel
CaCl₂: calcium chloride
CS: conditioned stimulus
DAPI: 4',6‐diamidino‐2‐phenylindole (nuclear stain)
DV: dorsal‐ventral (coordinate axis in stereotaxic surgery)
EE: environmental enrichment
EGFP: enhanced green fluorescent protein
EGTA: ethylene glycol‐bis(β‐aminoethyl ether)‐*N*,*N*,*N*′,*N*′‐tetraacetic acid (calcium chelator)
fAHP: fast afterhyperpolarization
GABA: gamma‐aminobutyric acid
GFP: green fluorescent protein
HEPES: 4‐(2‐hydroxyethyl)‐1‐piperazineethanesulfonic acid (buffering agent)
ID: inner diameter
mAHP: medium afterhyperpolarization
ML: medial‐lateral (coordinate axis in stereotaxic surgery)
mPFC: medial prefrontal cortex
NAc: nucleus accumbens
OD: outer diameter
PBS: phosphate‐buffered saline
PL: prelimbic cortex
PNN: perineuronal net
PV: parvalbumin (interneuron marker)
PVT: paraventricular nucleus of the thalamus
RI: random interval
ROI: region of interest
RRID: Research Resource Identifier (used for antibodies and software)
SEM: standard error of the mean
SH: standard housing
S‐aCSF: sucrose‐based artificial cerebrospinal fluid
SK: small conductance calcium‐activated potassium channel
SOM: somatostatin (interneuron marker)
US: unconditioned stimulus
VIP: vasoactive intestinal peptide (interneuron marker)
WinWCP: Windows Whole Cell Program (electrophysiology software)

## 1 Introduction

Signals or ‘cues’ associated with palatable foods (e.g., fast‐food advertisements) trigger food memory retrieval, but also responses such as food cravings that trigger unhealthy overeating (van den Akker et al. 2018). In laboratory animals, these cues invigorate food‐seeking behaviour, allowing us to conduct detailed neurobiological investigations into food cue reactivity. To date, such research has largely been performed to reveal ‘pro‐craving’ brain circuits that provoke cue reactivity, but there is a paucity of information about ‘anti‐craving’ circuits that suppress cue reactivity.

**FIGURE 1:**
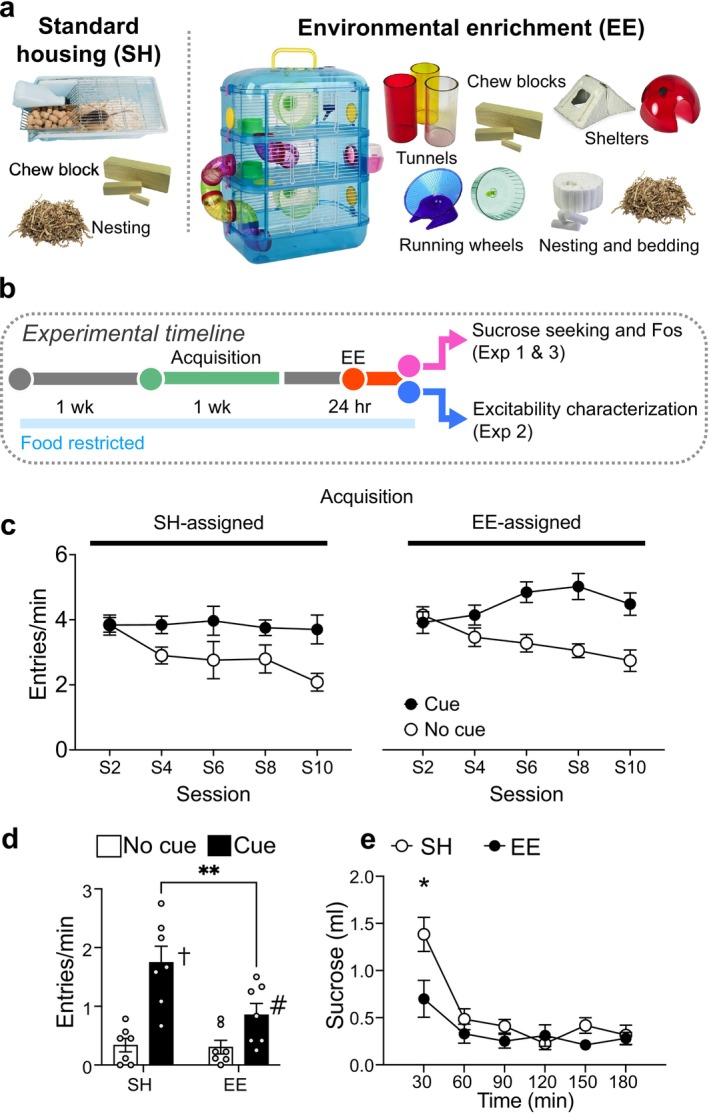
Experimental timelines and housing conditions. (a) Images of housing with EE and control SH. (b) Timeline. (c) Head entry rates into the magazine during the cue and no‐cue periods during acquisition for mice in the EE‐ and SH assigned conditions (*n* = 7/group). d EE selectively modulates cue‐evoked sucrose seeking. e EE attenuates sucrose consumption (*n* = 6–7/group). **p < 0.01 and *p < 0.05 against mice in SH condition. ^†^ *p* < 0.01 and #*p* < 0.05 against no cue condition within the EE or SH condition. All data are expressed as mean ± SEM. EE, environmental enrichment; SH, standard housing.

We and others have provided physical and cognitive stimulation to laboratory rodents by providing housing with environmental enrichment (EE) with the provision of larger cages with exercise wheels, tunnels and shelters, toys and multiple nesting materials (Nithianantharajah and Hannan 2006; Grimm and Sauter 2020; Solinas et al. 2020; Margetts‐Smith et al. 2021) (Figure 1a). One‐day EE exposure attenuates cue‐evoked sucrose seeking in both mice and rats (Grimm and Sauter 2020; Margetts‐Smith et al. 2021). Furthermore, EE attenuates the recruitment of sparse sets of activated neurons or ‘neuronal ensembles’ expressing the neuronal activity marker ‘Fos’ in the prelimbic area (PL) of the medial prefrontal cortex (mPFC), a brain structure that controls motivated actions (Dalley et al. 2004; Riga et al. 2014; Gourley and Taylor 2016). Specifically, EE reduces Fos in PL's deep layer neurons, which project to various subcortical, thalamic and striatal structures that coordinate appetitive behaviours (Berendse et al. 1992; Voorn et al. 2004; Vertes 2006; Riga et al. 2014; Anastasiades and Carter 2021).

Alterations in mPFC neuronal excitability and recruitment underlie the modulation of cue‐evoked food seeking (Brebner et al. 2020a; Brebner et al. 2020b). In the mPFC, both inhibitory interneurons that increase local inhibition and excitatory pyramidal cells that project to various subcortical structures, that subserve motivation and reward, orchestrate cue‐evoked reward‐seeking behaviours (Sparta et al. 2014; Otis et al. 2017; Brebner et al. 2020a; Brebner et al. 2020b). Here, we hypothesized that EE promotes the suppression of cue‐evoked food seeking through modulating the excitability of PL neurons and recruitment of PL projection neurons to the paraventricular nucleus of the thalamus (PVT) and nucleus accumbens core (NAc), which play key roles in processing cue‐related information and orchestrating reward seeking (Otis et al. 2017; Iglesias and Flagel 2021). Because women experience more food cravings and exhibit higher cortical activation following food cue exposure than men (Pelchat 1997; Zellner et al. 1999; Uher et al. 2006; Hallam et al. 2016), we focused our investigations on examining EE effects on modulating PL excitability and projection neuron recruitment in female mice.

## 2 Materials and Methods

### 2.1 Animals and Behavioural Procedures

Female C57BL/6J mice (Charles River, Margate, UK), aged 8–12 weeks, were housed 2–4 per cage under a 12‐h light/dark cycle (lights on at 7:00 AM), at 21 ± 1°C and 50 ± 5% humidity. One week prior to conditioning and throughout the study, mice were food restricted to 90% of ad libitum weight. All procedures complied with the UK Animals (Scientific Procedures) Act 1986.

Behavioural procedures followed previously described protocols (Margetts‐Smith et al. 2021). Mice were trained and tested in conditioning chambers with a syringe pump delivering 10% sucrose (US) into a magazine. The conditioned stimulus (CS or cue) was an auditory clicker.

Following magazine training, mice underwent 10 twice‐daily conditioning sessions, each with six 120‐s CS presentations separated by random‐interval (RI) 120‐s no cue intervals. During cue periods, ~15 μL of sucrose was delivered on an RI 30‐s schedule across the CS. Three to 7 days (Experiments 1 and 3) or 1–2 weeks (Experiment 2) after acquisition, mice were exposed to 1‐day EE or remained in standard housing (SH). EE cages were larger (40 × 26 × 53 cm), with three tiers, tunnels, a sleeping pod, two exercise wheels, varied nesting and bedding materials, plastic houses, chew bars and Lego bricks (Figure 1a) (Margetts‐Smith et al. 2021). SH consisted of standard cages (15.9 × 14 × 12.7 cm) with nesting material and a chew block. In Experiments 1 and 3, mice were tested under extinction conditions to assess cue‐evoked food seeking. In Experiment 2, neuronal excitability was assessed immediately after EE.

### 2.2 Adeno‐Associated Viruses Information

Experiment 2—For expression of mRuby in inhibitory neurons, we used AAVDJ/8/2‐mDlx‐mRuby (viral titre 6.9 × 10^12^ vg/mL; catalogue # v242‐DJ/8, Viral Vector Facility [VVF] of the Neuroscience Center Zurich [ZNZ], constructed using elements from Addgene, Watertown, Massachusetts, USA; mDlx‐HBB‐chI [#83900] and mRuby3 [#85146]). We diluted this virus 1/10 with sterile saline.

Experiment 3—For retrograde adeno‐associated virus (AAV) tracing, we used AAVrg‐hSyn‐EGFP (viral titre 7.0 × 10^12^ vg/mL; catalogue # 50465‐AAVrg, Addgene, Deposited by Bryan Roth) and AAVrg‐hSyn‐mCherry (viral titre 7.0 × 10^12^ vg/ml; catalogue # 114472‐AAVrg, Addgene, Deposited by Karl Deisseroth) diluted 1/10 with sterile saline.

### 2.3 Surgical Procedures

Mice (8–11 weeks old) were anaesthetized with isoflurane, placed in a stereotaxic frame (RWD Life Science, Shenzhen, China) and given preoperative analgesia (s.c. Meloxicam). A ~1‐cm scalp incision was made, and holes were drilled above infusion sites. A sterile glass pipette (Drummond, Broomall, Pennsylvania, USA) attached to a syringe pump (UMP3, World Precision Instruments, Sarasota, Florida, USA) was filled with virus and lowered to the target DV coordinate. A total of 500‐nL virus was infused at 100 nL/min (300 nL for retrograde tracing).

For mRuby, bilateral injections of AAVDJ/8/2‐mDlx‐mRuby were made into the PL (anterior–posterior [AP] + 1.8 mm, medial–lateral [ML] ± 0.35 mm, dorsal–ventral [DV] − 2.4 mm from bregma). For retrograde tracing, AAVrg‐GFP and AAVrg‐mCherry were injected unilaterally into NAc (AP + 1.2 mm, ML ± 1.1 mm, DV − 3.9 mm from brain surface) and PVT (20° angle, AP − 1.36 mm, ML ± 1.13 mm, DV − 3.3 mm from bregma). Mice recovered for 3–5 weeks before experiments commenced.

### 2.4 Immunohistochemistry and Fos Quantification

Fos staining was performed as previously described (Ziminski et al. 2017; Brebner et al. 2020b). Ninety minutes after the final test session, mice were anaesthetized with pentobarbital and transcardially perfused with 4% paraformaldehyde. Coronal sections (30 μm) were cut using a Leica CM1900 cryostat, including the PL (~AP 2.13–1.73) (Paxinos and Franklin 2001) and NAc and PVT injection sites.

Sections were incubated overnight at RT with 1:300 anti‐Fos primary antibody (cat# 2250, RRID:AB_2247211; Cell Signaling Technology, Danvers, Massachusetts, USA). The next day, slices were washed in PBS and incubated for 90 min with 1:300 anti‐rabbit Alexa 647 secondary antibody (Fos, cat# A‐21245, RRID:AB_2535813; Thermo Fisher, Waltham, Massachusetts, USA). DAPI (5 μM; cat# D‐9542; Sigma Aldrich) was used for nuclear staining. Slices were mounted (cat# 11562203; Fisher, UK) and coverslipped with Fluoromount G (cat# 00‐4958, RRID:SCR_015961; Invitrogen, Carlsbad, California, USA).

Immunofluorescence (Fos) and native fluorescence (GFP and mCherry) images of Fos, GFP and mCherry (Experiment 3) from left and right PL hemispheres (1–2 sections/animal, bregma 2.13–1.73) were captured using a 10× objective (N.A. 0.3; Olympus; Tokyo, Japan; and QI click camera; Qimaging; Surrey, British Columbia, Canada) on an Olympus Bx53 microscope. Fos + nuclei and regions of interests were quantified using iVision software (v4.0.15, RRID:SCR_014786; Biovision Technologies, Exton, PA, USA). Images were digitally merged from 10 Z‐stacks (10‐μm thickness) and normalised by contrast adjustment.

### 2.5 Ex Vivo Electrophysiology

Slice electrophysiology was performed as previously described (Ziminski et al. 2017; Brebner et al. 2020b). One to 2 weeks after the final acquisition session, mice were exposed to 1‐day EE or remained in control conditions. Brains were extracted and immersed in near‐freezing, oxygenated high‐sucrose artificial cerebrospinal fluid (SaCSF; mM: 60 sucrose, 87 NaCl, 2.5 KCl, 3 MgCl_2_, 0.5 CaCl_2_, 26 NaHCO_3_, 1.25 NaH_2_PO_4_·H_2_O, 10 D‐glucose), saturated with 95% O_2_ /5% CO_2_.

Coronal slices (250–300 μm) containing the prelimbic cortex (PL; ~AP 1.5–2.4 mm) were cut using a Leica VT1200S slicer. Slices were incubated in carbogenated aCSF at ~34°C for 30 min and then cooled to room temperature for 10–30 min before recordings. During recordings, slices were perfused at 2–3 mL/min with standard aCSF (mM: 125 NaCl, 2.5 KCl, 2 CaCl_2_, 1 MgCl_2_, 26 NaHCO_3_, 1 NaH_2_PO_4_·H_2_O, 10 D‐glucose), maintained at 30°C–32°C and bubbled with 95% O_2_/5% CO_2_.

**FIGURE 2:**
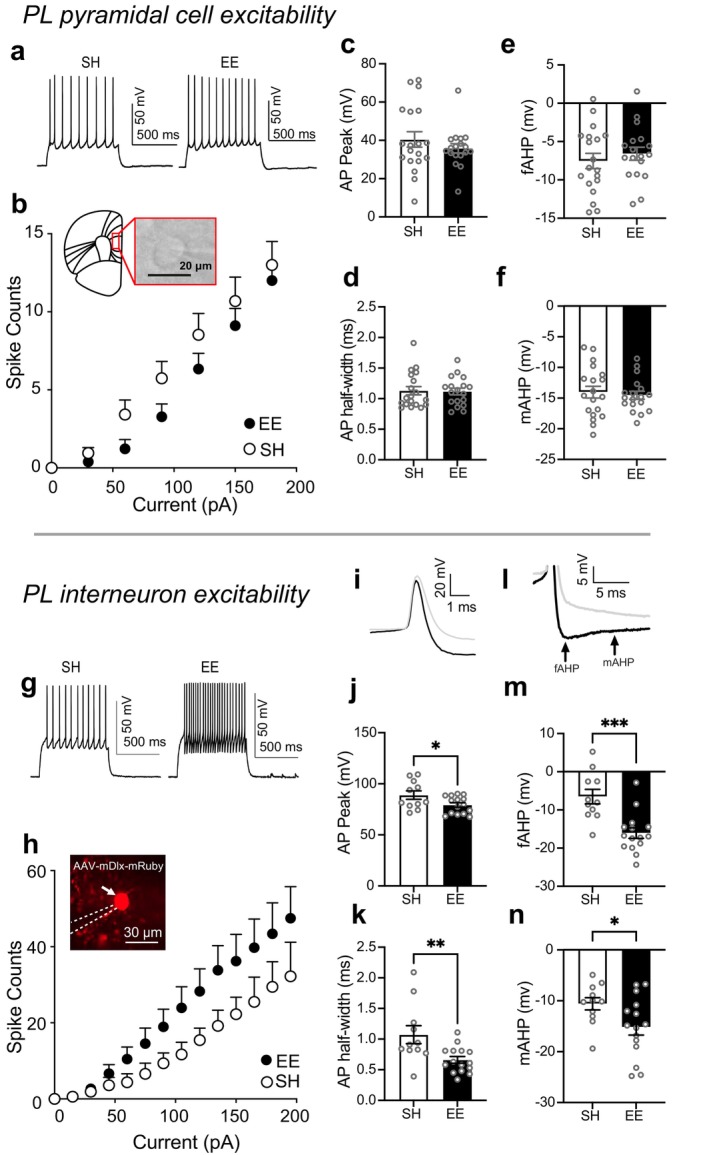
Environmental enrichment does not alter the intrinsic excitability of PL pyramidal cells (a–f) but modulates the excitability of PL inhibitory interneurons (g–n). (a) Representative current injection traces and (b) firing capacity of pyramidal cells from EE or SH (no EE) conditions (SH: *n* = 19/5, EE: *n* = 18/5), inset: representative pyramidal cell during recording. (c) AP, peak (d) half‐width, (e) fAHP, (f) mAHP from pyramidal cells from EE and SH conditions. (g) Representative current injection traces and (h) firing capacity of interneurons from EE and SH conditions (SH: *n* = 11/5, EE: *n* = 15/6), inset: representative mRuby+ interneuron during recording (indicated by white arrow). (i) Representative action potential and (l) AHP trace from interneurons from mice in EE (black line) and SH (grey line) conditions. (j) AP peak, (k) AP half‐width, (m) fAHP, and (n) mAHP of interneurons from mice in EE and SH conditions. *n* = total number of cells/total number of mice. *p < 0.05, **p < 0.01, ***p < 0.001 against mice in SH condition. All data are expressed as mean ± SEM. AHP, afterhyperpolarization; AP, action potential; EE, environmental enrichment; fAHP, fast afterhyperpolarization; mAHP, medium AHP; PL, prelimbic; SH, standard housing.

Whole‐cell recordings from PL layer V–VI pyramidal cells or interneurons (Experiment 2; Figure 2) were performed using glass pipettes (1.5 mm OD, 0.86 mm ID) for intrinsic excitability measurements. The intracellular solution contained the following (in mM): 125 K‐gluconate, 10 KCl, 2 MgCl_2_, 0.1 CaCl_2_, 10 HEPES, 1 EGTA, 2 Mg‐ATP and 0.2 Na‐GTP (pH 7.2–7.4). Pyramidal cells were identified by morphology and/or characteristic firing properties, as described in previous studies (Cao et al. 2009; Ziminski et al. 2017; Brebner et al. 2020b). Interneurons were identified via mRuby expression using 561‐nm excitation.

Data were collected with a Multiclamp 700B amplifier (Molecular Devices, San Jose, CA, USA; A/D board; PCI 6024E; National Instruments, Austin, TX, USA; and WinWCP Software [courtesy of Dr. John Dempster, University of Strathclyde, Glasgow, UK]).

(http://spider.science.strath.ac.uk/sipbs/software_ses.htm). Neurons were visualized with differential interference contrast using an Olympus BX51WI microscope attached to a Revolution XD spinning disk confocal system (252, Andor Technology, Belfast, UK) for fluorescence microscopy. Pyramidal cells and interneurons were held at −65 mV and −80 mV, respectively. Current clamp protocols consisted of 800‐ms injections: −60‐ to 730‐pA in 10‐pA steps for pyramidal cells and −60 to 450 pA in 5‐pA steps for interneurons. Membrane properties were analyzed using Easy Electrophysiology software (Easy Electrophysiology Ltd., London, UK).

### 2.6 Data Analysis

Behavioural testing, Fos quantification, electrophysiological recordings and analyses, as well as image processing, were all conducted by investigators blinded to group identity to minimize bias. All statistical tests were performed using Prism software (RRID:SCR_002798; GraphPad Software, San Diego, CA, USA). For all experiments, data were analyzed using multifactorial ANOVAs, followed by post hoc testing or pair‐wise comparisons using a two‐tailed *t* test. Group data are presented as mean ± SEM. Data points exceeding ±2 SDs from the mean were excluded as outliers.

## 3 Results

### 3.1 Experiment 1—EE Reduces Cue‐Evoked Sucrose Seeking and Sucrose Consumption

#### 3.1.1 Pavlovian Conditioning

We conditioned mice to associate an auditory cue with the delivery of 10% sucrose solution across 10 sessions (Figure 1b). Mice assigned to EE and SH conditions acquired a cue‐reward association at similar levels and exhibited selective head entry responding during the cue presentation period (auditory clicker) compared to the no‐cue period (session × EE × cue interaction, *F*_(9,126)_ = 0.733, *p* =0.6776; session × cue, *F*_(9,126)_ = 5.468; *p* < 0.0001; Figure 1c).

#### 3.1.2 Cue‐Evoked Sucrose Seeking and Sucrose Consumption Test

We assessed EE effects on cue‐evoked sucrose seeking under extinction conditions. Here, sucrose conditioned mice were either exposed to 1‐day EE or remained in SH (No EE controls) and underwent testing. Similar to our previous study (Margetts‐Smith et al. 2021), EE selectively reduced cue‐evoked sucrose seeking (EE × cue, *F*_(1,12)_ = 7.273, *p* < 0.05; main effects of EE, *F*_(1,12)_ = 4.857, *p* < 0.05 and cue, *F*_(1,12)_ = 37.90, *p* < 0.001; Figure 1d). An additional cohort of sucrose conditioned, EE‐exposed mice (or not exposed; SH mice) underwent sucrose consumption testing. EE significantly attenuated sucrose consumption (EE × time, *F*_(5,45)_ = 3.146, *p* < 0.05; main effect of EE, *F*_(1,9)_ = 5.967, *p* < 0.05; Figure 1e).

### 3.2 Experiment 2—EE Enhances Baseline PL Interneuron, but not Pyramidal Cell Excitability

We previously reported that EE reduced cue‐evoked Fos in PL (Margetts‐Smith et al. 2021). This finding suggests that EE modulates neuronal properties, such as intrinsic excitability that influences neuronal activity (Kourrich et al. 2015; Ziminski et al. 2017; Brebner et al. 2020b), even prior to cue exposure. Thus, we examined whether EE modulated ‘baseline’ (prior to test session) excitability in PL pyramidal cells and interneurons in layers V–VI, immediately following 1 d of EE.

#### 3.2.1 EE Effects on PL Pyramidal Cell Excitability

We analyzed the number of action potentials (APs) elicited in response to positive current step injections from pyramidal cells from mice in EE and SH conditions. EE did not modulate the baseline intrinsic excitability of PL pyramidal neurons as EE did not significantly modulate firing capacity (Figure 2a,b; EE condition × current; *F*_(20,700)_ = 1.23, *p* = 0.22) and active and passive membrane properties (Figure 2c–f; Table 1).

**TABLE 1:** Basic membrane properties from PL pyramidal cells following EE.

	Mean ± SEM	Mean ± SEM	
	SH	EE	*p*
Resting *V*_m_ (mV)	−66.16 ± 2.02	−68.33 ± 2.55	0.5059
Rheobase (pA)	74.74 ± 12.62	87.78 ± 9.10	0.4119
*R*i (mΩ)	187.4 ± 21.21	182.7 ± 18.38	0.8699
AP peak (mV)	42.26 ± 3.84	35.37 ± 1.14	0.1123
AP half‐width (ms)	1.09 ± 0.05	1.12 ± 0.05	0.6949
fAHP (mV)	−7.54 ± 0.99	−6.59 ± 0.85	0.4766
mAHP (mV)	−14.03 ± 0.96	−14.54 ± 0.65	0.6644

*Note:* Basic membrane properties from PL pyramidal cells from mice in EE and SH (no EE) conditions. All data are expressed as mean ± SEM.

Abbreviations: AP, action potential; fAHP, fast afterhyperpolarization; mAHP, medium afterhyperpolarization; *R*_i_, input resistance; *V*_m_, resting membrane potential.

#### 3.2.2 EE Effects on PL Interneuron Excitability

Mice were injected with AAV‐mDlx‐mRuby (Dimidschstein et al. 2016) in PL to virally express the red fluorescent protein ‘mRuby’ in GABAergic interneurons and we measured their excitability. EE significantly increased firing capacity (Figure 2g,h; EE condition × current, *F*_(87,1979)_ = 11.17, *p* < 0.001; main effect of EE, *F*_(1,24)_ = 5.94, *p <* 0.05; and current, *F*_(3.18,72.35)_ = 11.17, *p <* 0.001).

Additionally, we observed decreases in AP peak (Figure 2i,j; *t*_(24)_ = 2.233, *p* < 0.05), AP half‐width (Figure 2i,k; *t*_(24)_ = 2.930, *p* < 0.01), and in the fast and medium after hyperpolarization (Figure 2l,m; fAHP *t*_(24)_ = 4.221, *p* < 0.001; Figure 2l,n; mAHP *t*_(24)_ = 2.221, *p* < 0.05). No significant differences were observed for the resting membrane potential, rheobase and input resistance (Table 2). Hence, the enhanced firing observed in the EE condition is likely attributable to faster membrane repolarization, driven by increased afterhyperpolarizations (AHPs), which may reflect deinactivation of calcium‐modulated potassium channels.

**TABLE 2:** Basic membrane properties from PL interneurons following EE.

	Mean ± SEM	Mean ± SEM	
	SH	EE	*p*
Resting *V*_m_ (mV)	−65.41 ± 2.20	−69.67 ± 2.09	0.1815
Rheobase (pA)	64.55 ± 12.60	72.33 ± 16.94	0.7334
*R*_i_ (mΩ)	287.5 ± 40.90	312.40 ± 27.11	0.6002
AP peak (mV)	88.92 ± 4.10	79.26 ± 2.18 *	0.0351
AP half‐width (ms)	1.07 ± 0.15	0.66 ± 0.06 **	0.0073
fAHP (mV)	−6.51 ± 1.90	−16.08 ± 1.36 *	0.0003
mAHP (mV)	−10.59 ± 1.21	−15.20 ± 1.54 *	0.0361

*Note:* Basic membrane properties from mRuby+, PL interneurons from mice in EE and SH (no EE) conditions. All data are expressed as mean ± SEM.

Abbreviations: AP, action potential; fAHP, fast afterhyperpolarization; mAHP, medium afterhyperpolarization; *R*_i_, input resistance; *V*_m_, resting membrane potential.

* *p* < 0.05,

** *p* < 0.01.

### 3.3 Experiment 3—EE Attenuates Fos in PL → PVT, but not PL → NAc, Neurons

**FIGURE 3:**
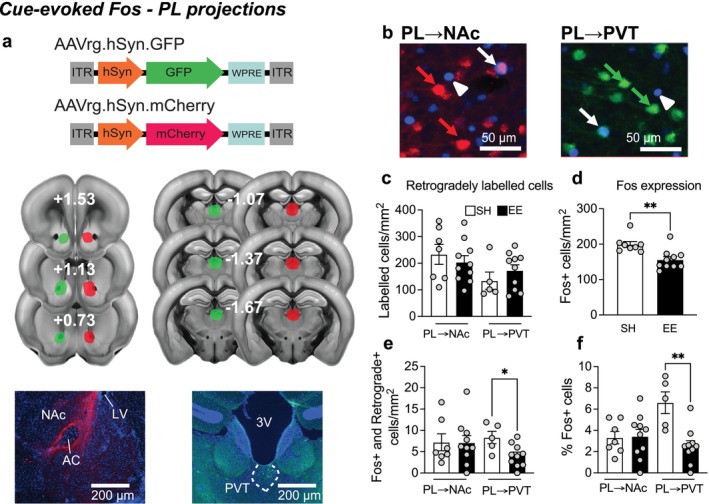
EE attenuated cue‐evoked Fos in PL → PVT, but not in PL → NAc neurons. (a) Overview of the retrograde AAVs used to express GFP and mCherry in the PL and their representative injection sites. (b) Representative images of Fos‐expressing cells (white arrowhead), PL → NAc (red arrow) and PL → PVT (green arrow) neurons that co‐express Fos (white arrow) (c) Similar levels of retrogradely labelled PL → NAc and PL → PVT neurons. (d) EE generally reduces Fos expression and e in PL → PVT, but not PL → NAc neurons. (f) Proportion of PL → NAc and PL → PVT neurons that express Fos. (c–f) PL → NAc (*n* = 7–10/group) and PL → PVT (*n* = 5–10/group). *p < 0.05 and **p < 0.01 against mice in SH condition. All data are expressed as mean ± SEM.

Using two retrograde AAVs, we labelled PL neurons projecting to the nucleus accumbens (NAc) and paraventricular thalamus (PVT) with GFP or mCherry in the same mice (counterbalanced). We then assessed Fos expression in these neuronal populations following EE. Viral injection sites are shown in Figure 3a and representative images of PL immunohistochemistry in Figure 3b. Retrograde labelling of PL → NAc and PL → PVT neurons was similar across mice in EE and SH (PL → NAc: (*t*_(15)_ = 0.685, *p* = 0.504), PL → PVT (*t*_(15)_ = 0.995, *p* = 0.338); Figure 3c). Similar to our previous study (Margetts‐Smith et al. 2021), EE reduced Fos (*t*_(16)_ = 3.496, *p* < 0.01; Figure 3d). Specifically, Fos expression in PL → PVT neurons was significantly lower in the EE compared to the SH condition (*t*_(13)_ = 2.729, *p* < 0.05), whereas there were no significant differences in Fos in PL → NAc neurons between EE and SH conditions (*t*_(15)_ = 0.042, *p* = 0.967; Figure 3e). Finally, the percentage of PL → PVT neurons expressing Fos was significantly lower in the EE compared to the SH condition (*U =* 2.0, *p* < 0.01), but no differences in the percentage of PL → NAc neurons expressing Fos between EE and SH conditions were observed (*U =* 34.5, *p* = 0.981, Figure 3f).

## 4 Discussion

Here, we investigated how EE attenuated cue‐evoked sucrose seeking via modulation of PL interneuron excitability and pyramidal cell recruitment. EE increased the baseline excitability within interneurons, but not pyramidal cells. Additionally, EE reduced recruitment of PL → PVT, but not PL → NAc, projection neurons. Our findings provide deeper insight into the modification of PL circuits relevant for ‘anti–food seeking’, consisting of PL interneuron excitability enhancement and corticothalamic suppression. We discuss below these EE‐mediated neuronal adaptations and their implications for modulating cue‐evoked sucrose seeking.

### 4.1 EE‐induced Enhancement of Interneuron, but not Pyramidal Cell, Excitability

EE increased interneuron excitability via enhancing firing capacity, thereby increasing PL's ability to exert more internal inhibitory control. In addition, associated with this enhancement were decreased AP half‐width and peak and increased mAHP and fAHP components of the AP. Because these components are influenced by voltage‐gated A‐type K^+^ channels and Ca^2+^‐dependent K^+^ channels (BK channels for fAHP and SK channels for mAHP), these alterations may reflect an alteration in function and/or number of these channels (Ishikawa et al. 2003; Villalobos et al. 2004; Kasten et al. 2007; Johnston et al. 2010; Wang et al. 2016). In general, K^+^ channels are associated with reducing excitability, but paradoxically, K^+^ channels can contribute to enhancing neuronal firing. For instance, the Kv3.3–3.4 subfamily of K^+^ channels that conduct A‐type K^+^ currents enhance firing rates by rapidly deinactivating voltage‐gated Na + channels, enabling faster recovery to resting conditions that allow subsequent neuronal firing (Kasten et al. 2007; Johnston et al. 2010; Jaffe and Brenner 2018). BK channels have also been implicated in this increased firing effect through their interaction with e.g., L‐type Ca^2+^ channels that enhance neuronal repolarization and decrease AP half‐width (Wang et al. 2016). Further neurophysiological investigations are required to elucidate the precise intrinsic factors underlying these alterations in interneurons following EE.

One factor contributing to enhanced excitability could be the increased formation of perineuronal nets (PNNs), a network of extracellular matrix (ECM) molecules on interneurons. Indeed, PNNs enhance interneuron excitability (Balmer 2016) and EE increases PNN intensity in mPFC interneurons (Slaker et al. 2016). PNNs typically surround parvalbumin‐(PV) expressing interneurons, which inhibit the activity of nearby pyramidal cells and interneurons and facilitate the suppression of sucrose seeking (Sparta et al. 2014). Hence, it is possible that EE enhances PV + interneuron excitability, which contributes to decreased Fos expression in PL → PVT neurons here.

Thus, in future studies, it would be paramount to identify whether PV + interneurons do indeed exhibit enhanced excitability, especially because we did not classify interneurons based on their phenotype. Indeed, several subtypes of interneurons exist, which are classified based on their neurochemical phenotypic markers such as PV, somatostatin (SOM) and vasointestinal peptide (VIP) and exhibit differences in intrinsic physiology (Anastasiades and Carter 2021).

Finally, we did not detect any changes in pyramidal cell excitability. One possibility for not detecting altered excitability is that we did not delineate between specific pyramidal cells, such as PL → PVT versus PL → NAc projection neurons that exhibited differential activity patterns following EE. Hence, subtle excitability changes may not have been detected. Future investigations should separately examine these neuronal populations to provide clearer insights into EE‐induced excitability adaptations.

### 4.2 EE Attenuated Recruitment of Corticothalamic, but not Corticoaccumbens, Projections

Related to the increased ability of PL interneurons to exert local inhibition, EE decreased recruitment of PL → PVT neurons. Initially, this decrease may seem at odds with (Otis et al. 2017), which showed that most PL → PVT neurons exhibited cue inhibition during sucrose conditioning and optogenetic stimulation of PL → PVT neurons suppressed sucrose seeking. However, there are procedural differences between our studies. Otis et al. recorded calcium activity in vivo in head‐fixed mice during the acquisition phase of sucrose conditioning, whereas we assessed Fos in freely moving mice under extinction conditions following EE. Importantly, Otis et al. found that most cue‐responsive PL → PVT neurons exhibited cue‐inhibitory responses, while a smaller subset showed cue excitation, highlighting functional heterogeneity. Optogenetic stimulation likely activated both subpopulations and suppressed sucrose seeking, as measured by anticipatory licking. Thus, it remains unclear whether behavioural suppression resulted from stimulating cue‐inhibited, cue‐excited, or both types of neurons.

Although Fos expression does not capture the heterogeneity of PL → PVT activity in vivo, it is reasonable to infer underlying activity dynamics based on its correlation with integrated calcium influx over time and longer calcium transients, rather than event frequency (Pettit et al. 2022). Therefore, EE could result in less sustained activity in a subpopulation of PL → PVT neurons over time during sucrose seeking. This decrease may reflect changes in encoding sucrose's diminished value after EE, as evidenced by reduced sucrose consumption. Here, EE represents a much larger reward than sucrose and resembles aspects of a devaluation manipulation (Sieburg et al. 2019). Indeed, reward devaluation decreases the proportion of putative PL pyramidal cells excited by sucrose cues (Niedringhaus and West 2022).

Alternatively, EE may recruit PL → PVT neurons with frequent activity events but without more sustained activity needed for Fos expression. Such neurons may represent a stable prelimbic ensemble that reliably encodes cue‐evoked responses (Grant et al. 2021). In support, although EE reduces sucrose seeking, cue‐selective behavioural responses remain evident (Figure 1d). Together, EE may attenuate sucrose seeking by reshaping PL → PVT ensemble dynamics rather than globally suppressing this pathway. Future studies using miniature endoscope imaging of PL → PVT ensem bles will be critical to disentangle contributions of cue‐excited and cue‐inhibited neurons to EE‐induced behavioural suppression.

EE did not alter PL → NAc recruitment despite its role in reward seeking (Gourley and Taylor 2016; Otis et al. 2017). However, this finding aligns with our previous study that EE did not decrease PL and NAc Fos expression (Margetts‐Smith et al. 2021). Furthermore, PL ensembles tagged during sucrose operant conditioning send only minor projections to the NAc (Visser et al. 2020). Hence, EE may attenuate sucrose seeking via preferentially modulating PL → PVT recruitment and brain structures targeted by the PVT, such as the central amygdala that contains neuronal ensembles that suppress cue‐evoked food seeking (Lay et al. 2023).

## 5 Summary and Future Directions

Here, we have extended our previous study (Margetts‐Smith et al. 2021) by revealing how EE dynamically enhanced PL's baseline interneuron excitability and attenuated recruitment of cue‐evoked corticothalamic neurons. Future studies need to delineate the precise interneuron populations, and the downstream neuronal targets modulated due to these cell‐type and circuit‐specific neuronal adaptations. We highlight how experience‐dependent plasticity within PL's inhibitory circuits may contribute to reduced PL output and suppression of food seeking, thereby revealing PL interneurons and PVT‐projecting neurons as potentially critical components of the brain's ‘anti‐craving’ circuitry.

## Author Contributions

**Kate Z. Peters:** formal analysis, investigation, methodology, project administration, software, visualization, writing – original draft, writing – review and editing. **Romarua Agbude:** formal analysis, investigation, writing – review and editing. **Oliver G. Steele:** formal analysis, investigation, supervision, writing – review and editing. **Nobuyoshi Suto:** formal analysis, investigation, writing – review and editing. **Eisuke Koya:** formal analysis, supervision, writing – original draft, writing – review and editing.

## Conflicts of Interest

The authors declare no conflicts of interest.

## Data Availability

Data relating to these experiments are available upon reasonable request from the corresponding author.
